# Psychometric properties of the Chinese version of the Hypoglycemia Fear SurveyII for patients with type 2 diabetes mellitus in a Chinese metropolis

**DOI:** 10.1371/journal.pone.0229562

**Published:** 2020-03-25

**Authors:** Chun Mu, Qiuling Xing, Yangkui Zhai

**Affiliations:** NHC Key Laboratory of Hormones and Development, Tianjin Key Laboratory of Metabolic Diseases, Chu Hisen-I Memorial Hospital & Tianjin Institute of Endocrinology, Tianjin Medical university, Tianjin, China; São Paulo State University (UNESP), BRAZIL

## Abstract

**Purpose:**

To evaluate the psychometric properties of the Chinese version of the Hypoglycemia Fear SurveyII (HFS-II) in patients with type 2 diabetes mellitus from Tianjin City.

**Methods:**

The original HFS-II was translated and adapted to Chinese.350 inpatients from five hospitals of Tianjin completed the Chinese HFS-II. We examined the validity (content and construct validity) and reliability (internal consistency and test-retest reliability) of the scale. Content validity was evaluated by the content validity index (CVI) and the average agreement CVI(S-CVI/Ave). The construct validity was assessed by exploratory factor analysis. Reliability was measured by intraclass correlation coefficient (ICC) and Cronbach’s alpha.

**Results:**

The mean age of the 350 patients was 55.5±9.3years. The CVI was 0.71~1.0 and S-CVI/Ave was 0.92 respectively. By exploratory factor analysis, four factors were extracted which accounted for 52.15% of the total variance in the 23-item scale. The Chinese HFS-II displayed good internal consistency (Cronbach’s alpha = 0.90) and test–retest reliability (ICC = 0.96).

**Conclusions:**

The Chinese version of HFSII had excellent psychometric properties and it could provide a useful tool for clinicians and nursing staff to assess the fear of hypoglycemia.

## Introduction

In China, the prevalence of diabetes has increased dramatically over the past 30 years[1]. According to the data of the International Diabetes Federation (IDF) in 2013, there were 98.4 million people with diabetes in China. The prevalence of diabetes in adults was 9.62% and type 2 diabetes accounted for over 90% [2]. The goal of diabetes management is to maintain normal blood glucose, however, the issue of hypoglycemia poses a threat to glycemic management[3]. Hypoglycemia, otherwise called a hypo, occurs when a patient’s blood glucose level falls below 3.9mmol/L, manifeted as weakness, trembling, sweating, light headedness, behaviour change and so on. For people with diabetes, when the balance isn’t quite right between medication and food and activity, a hypo may occur. Hypoglycemia unawareness is not preceded by warning signs mentioned above, which means a patient with low blood glucose concentration may not take any remedial action, becomes severely hypoglycemic. Severe hypoglycemia, at which point the patient is not able to independently treat themselves, causes mental confusion or coma, and even death if not treated in time[4]. The evidence of Diabetes Control and Complications Trail (DCCT) showed that intensive glycemic control not only reduced the occurrence of complications, but also increased the incidences of hypoglycemia[5]. Some studies showed that hypoglycemia increased risks of diabetes-related complications and it was related to fear and anxiety[6,7]. Frequent hypoglycemic events also posed severe psychological threats to patients who had emotional problems[8]. The experience of hypoglycemia induced the fear of hypoglycemia (FoH), which leads to poorer quality of life. The Hypoglycemia Fear Survey (HFS) was developed in 1987[9], which was revised in 2011 as the version of HFS-II. It consists of HFS-II-Worry Scale (HFSII-WS) and HFS-II-Behavior Scale (HFSII-BS) with a total of 33 items, which was widely used to measure worries and behaviors of FoH. Since it was first published, HFS-I, in its original form and subsequent revisions, has been used in 60 published studies and numerous clinical trials and translated into 50 countries, though not in China. HFS-I was commonly used in studies of type 2 diabetes. In addition, the authors have developed alternative versions of HFS-I for pediatric patients and type 1 diabetes. However, there are not much evidence for the reliability and validity of the HFS-II[10,11]. China has one third of the world's diabetic population, however, it currently lacks an assessment tool to measure FoH in type 2 diabetes patients. In this study, we translated the HFS-II into Chinese, adapted it to Chinese culture and validated the HFS-II in its initial version. The purpose of this study is to examine the psychometric properties of HFS-II for adult type2 diabetes in China.

## Methods

### Study design

This was a cultural adaptation and evaluation observational study of the psychometric properties (contend validity, construct validity and reliability) of an FoH questionnaire (the HFS-II) in a population with type 2 diabetes in Tianjin, China.

1. Translate the English version of HFS-II scale into Chinese (CHFS-II) and apply it to inpatients with type 2 diabetes in Tianjin City.
2. By convenience sampling, select volunteered patients by the inclusion and exclusion criteria. In the whole process of the study, the scale is completed by trained investigators, who will introduce the purpose of the study to the patient. The participants should fill out the informed consent form.
3. Evaluate the content validity, construct validity and reliability of CHFS-II.

### Participants

Convenience sampling was used to select 350 inpatients from December 2013 to May 2014. Five hospitals in Tianjin were included and each hospital had an independent Department of Endocrinology. Inclusion criteria were as follow: ≥18years old; diagnosed with type 2 diabetes >1 year; receiving ongoing treatment with oral antidiabetic agents or combined therapy (antidiabetic agents and insulin); normal cognitive function and consciousness; experienced hypoglycemia within the past 6 months. The patients with a history of mental illnesses or taking antipsychotic medication within the past 2 years were excluded. For factor analysis, the sample size should be 10 times to the number of the scale’s items(33 items)[12], so a total of 350 type 2 diabetes patients were recruited and 10% drop-out rate was allowed. Ethical approval was obtained from the Ethics Committee of Tianjin Medical University (NO.TMUhMEC2013050) and Chinese Clinical Trail Registry (NO.ChiCRT-ECS-13004095).

### Data collection

The purpose of the study was explained to participants before recruitment and written informed consent was obtained. Participants all completed the CHFS-II and the demographic survey questionnaire on the first day of admission. The CHFS-II was done again after 2 weeks and there was no missing participant in the followed-up interview, which means the drop-out rate was zero.

### Demographic survey questionnaire

The demographic questionnaire involves gender, age, education level, marital status, medical insurance, employment situation, medical history duration of diabetes, Body Mass Index (BMI), Hb_A1c_ and the frequency of hypoglycemia.

### The cultural adaptation of the Chinese version of hypoglycemia fear surveyII

The original HFS-II was developed by Cox DJ and Gonder-Frederick[9], which contains 33 items rated on a five-point Likert scale ranging from 0 (never) to 4 (always) with the total sum score 0–132. The higher the score, the greater the FoH. The HFS-II has shown a good test-retest reliability and Cronbach α coefficients in previous studies[11,13].

The cultural adaptation process of the scale was conducted based on the forward/backward translation method[14].Two Chinese native translators with master's degree in English and questionnaire translation experience independently translated it and then combined the two translations into one version. Then the new version was translated back into English by two American English teachers who are competent in Chinese. The researchers and four translators compared the newly translated versions to the original HFS-II, then the most appropriate expression for each item was selected and a methodologist supervised the whole translation process. After a series of discussion, the CHFS-II was completed.

### Statistical analysis

All statistical analysis was performed using SPSS (version 18.0). The demographic characteristics were analyzed using descriptive statistics. The content validity was evaluated by the content validity index (CVI) and the average agreement CVI(S-CVI/Ave). The CHFS-II was emailed to an expert panel for evaluation. The experts panel consists of 3 Chief endocrinologists with over 10 years of clinical experience, 3 nurses with over 10 years of experience in diabetic care and 1 type 2 diabetes patient diagnosed over 5 years and experienced hypoglycemia in the last 6 months. A four-point rating scale was used to calculate the CVI: 1 = no relevance, 2 = low relevance, 3 = strong relevance, 4 = very strong relevance. There are two kinds of CVI: the item-level CVIs (I-CVIs) and the scale-level CVI(S-CVI)[15]. The I-CVI = the number of experts who rated the item 3 or 4 **/**the number of experts, which reflects the correlation of each item to the scale. According to Lynn’s study, the I-CVI value of each item≥ 0.78 is considered acceptable[16]. The S-CVI/AVE is the average of all I-CVIs of the scale[17] and according the Waltz’s study[18], the S-CVI/AVE value reaching 0.90 is considered valid.

The construct validity was evaluated by exploratory factor analysis (EFA). The sample suitability was confirmed by the Kaiser-Meyer-Olkin (KMO) measure and Barlett’s Sphericity Test. Generally, Kaiser-Meyer-Olkin value reaching 0.8 and Chi-square value reaching significant level (P<0.001) indicates acceptable fit[12]. After the suitability had been checked, the main factors were extracted and varimax rotation was performed using the Principal Component Analysis of EFA. A minimum eigen value of 1 was assigned as extraction criterion and item loading higher than 0.3 was the criterion to have a successful loading on one factor. If items cross-loaded on two factors, one item factor was deleted. Pearson correlation coefficient was used to examine the factor correlation matrix. The test-retest reliability was evaluated by the intraclass correlation coefficient (ICC). A two-way random effects model with a single absolute agreement was used[18]. An ICC value≥ 0.75 means an excellent reliability[19]. Internal consistency was measured with Cronbach’s alpha. The value of Cronbach’s alpha>0.7 indicated the internal consistency acceptable[12].

## Results

Demographic and clinical characteristics of participants were presented in **Table 1**. 350 inpatients with type 2 diabetes were included. The Mean (SD) age of the participants was 55.5(9.3) years and 53.1% was male. The Mean (SD) duration of diabetes was 11.5(7.0) years and the average Hb_A1c_ was 8.6(1.7)%.

**Table 1:** Demographic and clinical characteristics of participant(N = 350).

Characteristics	N	(%)	Mean(SD)
Age(yrs)	350		55.5(9.3)
Gender			
male	186	53.1	
female	164	46.9	
Education level			
Elementary or lower	47	13.4	
Junior high	95	27.1	
Senior high	94	26.9	
College or above	114	32.6	
Marital status			
Single	16	4.6	
Married	306	87.4	
Divorced	8	2.3	
Widowed	20	5.7	
Employment status			
Emplolyed	123	35.1	
Unemployed	227	64.9	
Medical insurance			
Yes	312	89.1	
No	38	10.9	
BMI	350		26(3.7)
Duration	350		11.5(7.0)
HbA1c	350		8.6(1.7)
Frequency of hypoglycemia			
1–2 times in six months	181	51.7	
3–6 times in six months	89	25.5	
More than 1 time per month	69	19.7	
More than 1 time per week	11	3.1	

HbA1c = glycated hemoglobins; BMI = Body Mass Index N = number; (%) = Percent; SD = standard deviation.

According to the experts panel’s ratings of all 33 items, 30 items were rated 3(strong relevance) or 4(very strong relevance) by over fives experts out of seven except item 2, 17 and 24. The content validity index (I-CVIs) of the each item ranged from 0.71–1.0. All items on the scale had excellent evaluation (I-CVIs≥0.78) except item 2, 17 and 24(I-CVIs < 0.78). The S-CVI/Ave of the scale was 0.92. The probability of random correlation coefficient (Pc) of item 2, 17 and 24 was >0.05. We conducted a principle factor analysis by Quartimax rotation for the CHFS-II. Following the exploratory factor analysis and confirmation of the sample suitability of the 33 items of CHFS-II, the number of items was 23. The results of the factor analysis and factor correlation were presented in **Table 2**. Four factors were extracted which accounted for 52.15% of the total variance in the 23-item scale. Factor 1 had an eigen value of 7.35, which accounted for 31.94% of the response variance; factor 2 had an eigen value of 1.93(8.4% of the response variance); factor 3 had an eigen value of 1.4 (6% of the response variance) and factor 4 had an eigen value of 1.32 (5.7% of the response variance).

**Table 2:** Exploratory factor analysis loading in pattern and structure matrix(N = 350).

Item	Factor1 (worry)	Factor 2 (avoid)	Factor 3 (Embarrassing)	Factor 4 (behaviour)
W1	.578			
W3	.699			
W5	.759			
W7	.648			
W8	.730			
W10	.819			
W12	.601			
W13	.498			
W14	.801			
W15	.625			
W16	.491			
W17	.562			
B6		.660		
B7		.576		
B8		.804		
B9		.776		
B10		.346		
W4			.686	
W6			.501	
W11			.774	
B3				.433
B4				.695
B15				.554
Kaiser-Meyer-Olkin (Barlett’s Test of Sphericity) 0.88(p<0.001)				
Factor correlation matrix^a^				
Worry	-	0.482	0.378	0.538
Avoid	0.482	-	0.304	0.409
Embarrassing	0.538	0.304	-	0.233
Behaviour	0.538	0.409	0.233	-
CHFS-II	0.940	0.612	0.699	0.579

^a^ express as Pearson r.

As for Pearson correlation coefficient, the value of r>0.6 means high correlation, 0.3<r<0.6 means moderate correlation and r<0.3 means low correlation[20]. The factor correlation matrix showed strong to moderate correlation between four factors and CHFSII (r = 0.94;0.612;0.699;0.579) and moderate correlations among all factors except the correlation between embarrassing emotions and behavior change (r = 0.233). The result were showed in Table 3.

**Table 3:** Internal consistency and test-retest of reliability (N = 350).

	Cronbach’s alpha	ICC (95% CI)
CHFS-II	0.90	0.96 (0.93–0.98)
Worry	0.89	0.900(0.81–0.95)
Avoid	0.78	0.959(0.92–0.98)
Embarrassing	0.71	0.961(0.92–0.98)
Behaviour	0.63	0.894(0.80–0.95)

ICC, Intraclass correlation coefficient; 95%CI,95% Confidence Interval.

**Table 3** presented the results of internal consistency and test-retest reliability. Internal consistency was evaluated by the Cronbach's alpha coefficient, which was 0.90 for the whole scale and 0.63–0.89 for the four factors. The ICC value showed satisfactory reliability for the whole scale and each factors (ICC = 0.96; 95%IC = 0.93–0.98).

## Discussion

The HFS-II has been translated and adapted in many western countries as a valid tool to measure the behaviours and worries relating to the fear of hypoglycemia[13,22]. Regular assessment of FoH is crucial to Chinese type 2 diabetes since hypoglycemia was not always recognized by clinicians. Although the HFS-II has good reliability and validity, the factor structure of the scale is still controversial and needs future validation.

The Chinese version HFS-II has an excellent content validity in our study. According to the previous study, the I-CVI value ≥0.78 was considered acceptable[15]. There were three items (2, 17, 24) did not reach the criterion of excellence and the I-CVI was 0.71. For item 2 (Tried to keep my blood sugar above 8.3mmol/L or 150mg/dl), the patient and experts panel explained that most people with diabetes would keep their blood sugar at a normal level but not a high level for the prevention of hypoglycemia. For item 17 (Not having food, fruit, or juice available), most Chinese diabetic patients used to carrying candies when they go out and clinicians told them to take some snacks to cope with unexpected hypoglycemia, which should not be a reason for FoH. For item 24 (Having a hypoglycemic episode while driving), most type 2 diabetic patients are senior citizens and the mean age of participants in our study was 55.5(9.3) years, who would prefer riding bicycle or taking public transportion to driving in Tianjin City. However, it has an excellent internal consistency (Chronbach’s alpha = 0.90) and test-retest reliability (intraclass correlation coefficient = 0.96). These results were comparable to the study of Type 1 diabetes by Gonder-Frederick[10].

Only Twelve HFS-B items fit well with proper ranges of infit values (0.64–1.28) in the original study of DJ Cox, and part of the HFS-B items did not fit very well. So in our study, we used the EFA to re-analyze the structure of the scale. EFA is a statistical method to explore the components or subscales of the scale and analyze its construct structure and validity. In DJ Cox’s study, the scale is a 2-factor structure while Gonder-Frederick analyzed the scale and extracted a 3-factor structure [9.10]. In our study, we extracted a four-factor structure. This result is inconsistent with the results of the factor analysis by DJ Cox and Gonder-Frederick. Factor 1 consists of twelve items of”worries about hypoglycemia” such as item 5 “Make sure I have someone with me when I go out”; Factor 2 mainly consists of four items concerning about “avoid” such as item 7 “Limited my driving (car, truck or bicycle)”; factors 3 and 4 consists of two main items of “embarrassing emotion” and “behavior change”, such as item 21 “Appearing stupid or drunk” and item3 “Reduced my insulin when my blood sugar was low”. Some items had to be excluded because of low loading on any of the four factors (<0.3).

According to the loading threshold (>0.3), ten items of the original scales were deleted and 23 items were retained for the selected scale. Seven of the ten excluded items from the HFS-II-BS have always been controvercial and needed further validation. A previous study had shown some items of HFSII-BS had a poor loading, which was similar to our study[10]. To our knowledge, this is the first time a four-factor structure has been extracted and we divided the HFSII-WS into two distinct subscales reflecting worries about hypoglycemia and embarrassment due to the occurrance of hypoglycemia. The reason for the outcome of the new factor “Embarrassing” may the different language environment and culture background between the West and the East. In “confucian culture”, the courtesy is more inclined to be the individual's behavior and expression in society. In the case of hypoglycemia, the patient's poor performance is embarrassing. For example, item 26“Getting a bad evaluation or being criticized”, which just makes Chinese patients embarrassed but not worried being judged. Another possible explanation is the difference in sample size between our study and previous ones.

In the correlation matrix of CHFS-II, the four extracted factors all showed moderate correlation, which confirmed the interrelationship between different aspects of FoH. The intraclass correlation coefficient of each factor was excellent and adequate. The Cronbach’s alpha of all factors were acceptable except factor “behaviour” (0.63). Also the correlation between factor “behaviour” and “embarrassing” is low (0.233). The correlation coefficients of the four factors extracted by exploratory factor analysis were 0.233–0.538, and the “factor 4” and “factor 3” were weakly correlated due to poor internal consistency and lower factor loading. The reason may be related to the multi-dimensional content of the item. The original HFSII-BS contained items about correct diabetes management behavior like item“Reduced my insulin when my blood sugar was low” and item of inappropriate behavior to avoid hypoglycemia like item“Limited my exercise/physical activity”. These two different kinds of “behavior” items adopted the same scoring method [13], so CHFSII-BS remains to be further analyzed and revised. The decreased internal consistence was also supported by the original study of Cox DJ[21] and Anderbro[22]. In Gonder-Frederick’s study, the explanation was that the factor measured both appropriate and inappropriate behaviors aimed at avoiding hypoglycemia[10].

As a result, the psychometric properties evaluation suggested a four-factor model could be used. Our study demonstrated the CHFS-II had excellent reliability, content validity and reasonable factor structure in type 2 diabetes mellitus for assessing FoH in Tianjin.

We have to admit that our study has limitations due to the small sample of participants recruited in one northern industrial city of China. Due to the diversity of ethinic groups and culture in China, further multi-center studies should be conducted throughout this country to better prove our study. The HFS-II is a reliable and valid measure of FOH. By making HFS-II available in China, the clinicians could alter the patients’ treatment plan by assessing the FoH and nurses could provide better diabetic education to those suffer from hypoglycemia.

## Supporting information

S1 Data (DOC)
