# Supplementary material for: Psychometric properties of the Chinese version of the Hypoglycemia Fear SurveyII for patients with type 2 diabetes mellitus in a Chinese metropolis
Source: PLoS One. 2020 Mar 25;15(3):e0229562. doi: 10.1371/journal.pone.0229562 (PMC7096186; doi:10.1371/journal.pone.0229562)
Supplement: S1 Data — (DOC) [file pone.0229562.s001.doc]

附录

**附录一 中文版低血糖恐惧调查表（CHFSII）**

| **中文版成人低血糖恐惧调查表（CHFSII）** |
| --- |
| **一、 行为量表：**以下条目是糖尿病患者为了避免低血糖及低血糖的后果而产生的一些行为。请您从下列5个选项中选出一个能正确描述您在过去6个月中为避免低血糖及其后果所发生的行为的那个选项。（请不要漏填）  **填表说明：**所有题目均共用答案，请在对应的选项下划“×”，每题限选一个答案。 |
| 为了避免发生低血糖和它对我的影响，我会： 从没有 很少有 有时 经常这样 总是这样 |
| 1. 当我一感到有低血糖的迹象，我就吃些东西 □ □ □ □ □ |
| 2. 让我的空腹血糖保持在8mmol/L以上 □ □ □ □ □ |
| 3. 当我血糖降低的时候，减少胰岛素或药物的剂量 □ □ □ □ □ |
| 1. 增加血糖监测次数 □ □ □ □ □ |
| 1. 保证外出有人陪同 □ □ □ □ □ |
| 1. 减少出游或旅行 □ □ □ □ □ |
| 1. 限制驾驶（汽车、自行车） □ □ □ □ □ |
| 1. 避免走亲访友 □ □ □ □ □ |
| 1. 因害怕出现低血糖而不得不呆在家中 □ □ □ □ □ |
| 1. 限制运动/体力活动 □ □ □ □ □ |
| 1. 确保周围有人陪同 □ □ □ □ □ |
| 1. 为避免低血糖而随身携带糖块或碳水化合物 □ □ □ □ □ |
| 1. 参加一些活动的时候（如婚礼），保持血糖 □ □ □ □ □   高于平时 |
| 1. 做重要的事情时（工作），保持血糖高于平时 □ □ □ □ □ |
| 1. 让其他人无论白天或晚上关注我几次 □ □ □ □ □ |
| **二、 顾虑量表：**以下条目是描述糖尿病患者对于低血糖的顾虑。请仔细阅读每个条目。请您选出每个条目中能正确描述您在过去6个月中对低血糖所存在的顾虑的那个选项。  **填表说明：**所有题目均共用答案，请在对应的选项下划“×”，每题限选一个答案。 |
| 因为我的血糖可能下降，我担心： 从没有 很少有 有时 经常这样 总是这样 |
| 16、当我要发生低血糖的时候，自己没意识到 □ □ □ □ □ |
| 17、身边没有触手可及的食物、水果、饮料 □ □ □ □ □ |
| 18、在公共场合晕倒 □ □ □ □ □ |
| 19、在社交场所让自己或朋友感到尴尬 □ □ □ □ □ |
| 20、一个人的时候发生低血糖 □ □ □ □ □ |
| 1. 显得很愚蠢或醉态 □ □ □ □ □ |
| 22、失去控制 □ □ □ □ □ |
| 23、在发生低血糖的时候，周围没人帮助 □ □ □ □ □ |
| 24、在开车/骑车的时候发生低血糖 □ □ □ □ □ |
| 25、出现过失或意外 □ □ □ □ □ |
| 26、受到别人不好的评论或议论 □ □ □ □ □ |
| 27、低血糖时影响我的正确判断 □ □ □ □ □ |
| 28、感到头晕眼花 □ □ □ □ □ |
| 29、意外弄伤自己或他人 □ □ □ □ □ |
| 30、对自己的躯体或健康造成永久性伤害 □ □ □ □ □ |
| 31、低血糖的发生会打乱我正在做的某些重要的事情 □ □ □ □ □ |
| 32、睡眠中发生低血糖 □ □ □ □ □ |
| 33、突然感到烦躁不安并难以平静下来 □ □ □ □ □ |
